# Renal effects of treatment with a TLR4 inhibitor in conscious septic sheep

**DOI:** 10.1186/s13054-014-0488-y

**Published:** 2014-09-03

**Authors:** Johan Fenhammar, Mats Rundgren, Kjell Hultenby, Jakob Forestier, Micael Taavo, Ellinor Kenne, Eddie Weitzberg, Stefan Eriksson, Volkan Ozenci, Annika Wernerson, Robert Frithiof

**Affiliations:** Department of Anesthesia and Intensive Care, Karolinska University Hospital, Department of Clinical Science Intervention and Technology, Karolinska Institutet, Hälsovägen 13, 14157 Huddinge, Sweden; Department of Physiology and Pharmacology, Karolinska Institutet, von Eulers väg 8, 17177 Stockholm, Sweden; Department of Laboratory Medicine, Division of KFC, Karolinska Institutet, Hälsovägen 13, 14157 Huddinge, Sweden; Department of Surgical Sciences, Section of Anaesthesiology & Intensive Care, Uppsala University, Sjukhusvägen 1, 75185 Uppsala, Sweden; Department of Molecular Medicine and Surgery, Karolinska Institutet, Karolinska vägen 1, 17176 Stockholm, Sweden; Center of Molecular Medicine, Karolinska University Hospital, Karolinska vägen 1, 17176 Stockholm, Sweden; Division of Clinical Microbiology, Karolinska Institutet, Karolinska University Hospital, Hälsovägen 13, 14157 Huddinge, Sweden; Department of Clinical Science Intervention and Technology, Division of Renal Medicine, Karolinska Institutet, Hälsovägen 13, 14157 Huddinge, Sweden

## Abstract

**Introduction:**

Acute kidney injury (AKI) is a common and feared complication of sepsis. The pathogenesis of sepsis-induced AKI is largely unknown, and therapeutic interventions are mainly supportive. In the present study, we tested the hypothesis that pharmacological inhibition of Toll-like receptor 4 (TLR4) would improve renal function and reduce renal damage in experimental sepsis, even after AKI had already developed.

**Methods:**

Sheep were surgically instrumented and subjected to a 36-hour intravenous infusion of live *Escherichia coli*. After 12 hours, they were randomized to treatment with a selective TLR4 inhibitor (TAK-242) or vehicle.

**Results:**

The *E. coli* caused normotensive sepsis characterized by fever, increased cardiac index, hyperlactemia, oliguria, and decreased creatinine clearance. TAK-242 significantly improved creatinine clearance and urine output. The increase in N-acetyl-beta-D-glucosaminidas, a marker of tubular damage, was attenuated. Furthermore, TAK-242 reduced the renal neutrophil accumulation and glomerular endothelial swelling caused by sepsis. These effects were independent of changes in renal artery blood flow and renal microvascular perfusion in both cortex and medulla. TAK-242 had no effect per se on the measured parameters.

**Conclusions:**

These results show that treatment with a TLR4 inhibitor is able to reverse a manifest impairment in renal function caused by sepsis. In addition, the results provide evidence that the mechanism underlying the effect of TAK-242 on renal function does not involve improved macro-circulation or micro-circulation, enhanced renal oxygen delivery, or attenuation of tubular necrosis. TLR4-mediated inflammation resulting in glomerular endothelial swelling may be an important part of the pathogenesis underlying Gram-negative septic acute kidney injury.

**Electronic supplementary material:**

The online version of this article (doi:10.1186/s13054-014-0488-y) contains supplementary material, which is available to authorized users.

## Introduction

Acute kidney injury (AKI) is an important contributor to morbidity and mortality among hospitalized patients. Sepsis is the leading cause of AKI in the critically ill patient, and currently no effective treatment exists [1]. Septic AKI is generally believed to be due to regional hypoperfusion causing renal ischemia [2]. However, recent experimental reports indicate that AKI may develop even though renal blood flow and blood pressure remain within physiological limits [3-5]. Thus, it is possible that the pathogenesis of septic AKI is complicated by factors other than ischemia. In line with this, we wanted to investigate whether an important receptor for initiating inflammation, the Toll-like receptor 4 (TLR4), participates in the pathophysiology of *Escherichia coli*-induced AKI. TLRs have been identified as pivotal mediators in host defense as they are crucial in pathogen recognition and activation of the immune response [6,7]. The main ligand for TLR4 is lipopolysaccharide (LPS), a component of the cell membrane of Gram-negative bacteria [8-10]. When LPS binds to TLR4, an inflammatory response is initiated via production and release of cytokines as well as stimulation of inflammatory cells [11]. Targeting TLR4 with antibodies or specific inhibitors has shown beneficial effects by reducing mortality in experimental models of Gram-negative sepsis [12-17]. Initial clinical trials also showed beneficial effects of blocking TLR4 in sepsis [18], but in larger follow-up investigations, survival was not improved [19,20]. However, the renal effects of TLR4 antagonism are incompletely investigated, and there are reasons to believe that TLR4 may be an important mediator of sepsis-induced AKI. For example, the expression of TLR4 in renal tubules, glomeruli, and peri-tubular capillaries is increased after sepsis [21], and mice deficient in TLR4 have a reduced increase in blood urea nitrogen (BUN) when subjected to LPS [22]. Our group recently showed that pre-treatment with TAK-242 attenuated oliguria and reduced creatinine clearance in LPS-induced hyperdynamic shock [3]. However, although the aforementioned results are promising, the potential of TLR4 modulation during sepsis and septic renal failure, as well as mechanisms of action, is still largely unknown.

One of the main objectives with the current study was to investigate whether TLR4 inhibition is effective in attenuating or reversing renal dysfunction even after sepsis has developed. Therefore, TAK-242 was administered 12 hours into normotensive ovine sepsis caused by an intravenous live *E. coli* infusion, and the effect on renal function was followed for an additional 24 hours. To further explore the hypothesis that septic AKI is due to regional blood flow restriction, total renal blood flow and cortical and medullary microcirculation were continuously measured. Signs of ischemia were repeatedly monitored by microdialysis in the cortex and medulla. It is highly debatable whether septic AKI is associated with renal histopathology, and studies investigating structural damage to the kidney in long-term animal model of sepsis are lacking [23]. Thus, both light and electron microscopy were used to analyze renal samples taken at the end of the 36-hour study period with regard to local injury and leukocyte infiltration.

## Materials and methods

For detailed description of methods, please refer to the online supplemental material (Additional file 1). The experiments conform to the guidelines laid out in the Guide for the Care and Use of Laboratory Animals (National Academy of Science). The Regional Ethics Committee for Experiments in Animals, Stockholm, Sweden, approved the study in advance (N285/08).

### Surgical preparation and study protocol

Twenty-seven adult Texel crossbred ewes were included in the study. Twenty-four of these sheep were anesthetized and prepared with catheters in the right carotid artery, the pulmonary artery, and the right jugular vein. For renal hemodynamic measurements, an ultrasonic flow probe was placed around the left renal artery, and two laser Doppler flow probes, one cortical and one medullar, were sutured on the left kidney. Intrarenal metabolism was studied by microdialysis catheters in the cortex and in the medulla, respectively. A urinary retention catheter was inserted into the bladder for urine sampling. After a post-surgical recovery period of 12 to 18 hours, the experiments commenced with the animals being conscious and placed in a pen. Sepsis was induced by an intravenous infusion of live *E. coli* bacteria (bolus of 3.9 × 10^9^ colony-forming units followed by an infusion of 6.0 × 10^9^/mL colony-forming units, starting at a rate of 0.2 mL**/**hour). The infusion rate was increased stepwise every 6 hours until reaching 4 mL/hour after 30 hours. After 12 hours of sepsis, sheep were randomized to receive a bolus dose (2 mg/kg) followed by a continuous infusion (4 mg/kg per 24 hours) of either the selective TLR4 inhibitor TAK-242 (10 mg/mL) (n = 7) or vehicle (n = 7). The treatment was blinded to the investigators, and the content of the infusions was revealed only after the experiments were performed. To exclude that surgery per se had a major impact on the results obtained in the TAK-242 and vehicle groups, an additional sheep served as time control. This included surgical preparation and recovery and monitoring for 36 hours but no *E. coli* infusion or treatment.

To investigate whether TAK-242 had any effect on renal function per se, an additional three sheep were surgically prepared and, in a cross-over design, subjected to treatment with either TAK-242 or vehicle. The treatment was initiated by a bolus dose (2 mg/kg) followed by a continuous infusion (2 mg/kg per 12 hours). No *E. coli* was administered.

Besides the intravenous fluids given post-surgery, fluid volume support was administered as Ringer’s Acetate solution (B. Braun Melsungen, AG, Melsungen, Germany) intravenously at 1 mL/kg per hour and started 6 hours before the infusion of live *E. coli* bacteria. Blood samples (approximately 20 mL of venous blood and 1 mL of arterial blood) were drawn at baseline and every 6 hours after commencement of sepsis. Urinary output was measured and urine samples were collected every second hour. After 36 hours of sepsis, animals were deeply anaesthetized with sodium thiopental and terminated by an overdose of potassium chloride. The kidney was rapidly harvested and prepared for histological evaluation. The position of the laser Doppler probes and microdialysis catheters was confirmed visually by opening the kidney post-mortem. Renal biopsies were immediately frozen and stored at −70°C. If the animal was judged to be severely ill and in distress, it was euthanized prior to the end of the protocol.

### Histological evaluation of renal biopsies

Small pieces of renal tissue were prepared and evaluated by light and transmission electron microscopy. In addition, some sections were stained with anti-myeloperoxidase antibody for easy quantification of polymorphonuclear leukocyte (PMN) cells in the glomeruli, the interstitium, and the peritubular capillaries.

### Statistical analysis

Cardiovascular parameters were averaged off-line. Creatinine clearance was calculated as (urine flow × urine creatinine concentration)/plasma creatinine concentration. Cardiac output was indexed to body surface area (0.09 × body weight^0.67^).

All statistical calculations were performed by using Statistica 8.0 (Statsoft Inc., Tulsa, OK, USA), and the graphs were created with Sigma Plot 11.0 (SPSS Inc., Chicago, IL, USA). Data are expressed as means ± standard deviation of the mean or as mean and 95% confidence interval. Urine production, fractional sodium excretion (FENa), and urinary-N-acetyl-beta-D-glucosaminidase (U-NAG) values were transformed to follow a normal distribution by taking the logarithm of the raw data. Changes in parameters over time were analyzed according to a two-way repeated measures analysis of variance (ANOVA), with time as within effects and treatment (control/TAK-242) as between effects. If there was a significant interaction between time × treatment, an additional one-way repeated measures ANOVA was performed for each treatment to investigate whether that group changed significantly over time. The result of this analysis is not displayed in the figures but is referred to in the Results section. The significance level was set at a *P* value of not more than 0.05. Mann-Whitney *U* test was used to evaluate difference in PMN count and histological scores between treatments.

## Results

Nine out of twenty-three sheep subjected to *E. coli* had to be euthanized before completion of the protocol: five before treatment was started as well as two TAK-242-treated animals and two vehicle-treated animals between 12 and 14 hours of sepsis. These sheep are not included in the data presented in the figures/tables or the statistical analyses. No effect of surgery per se was seen in the sheep that was sham-operated (Additional file 2: Table S3).

### *E. coli* infusion caused hyperdynamic sepsis

**Figure 1:**
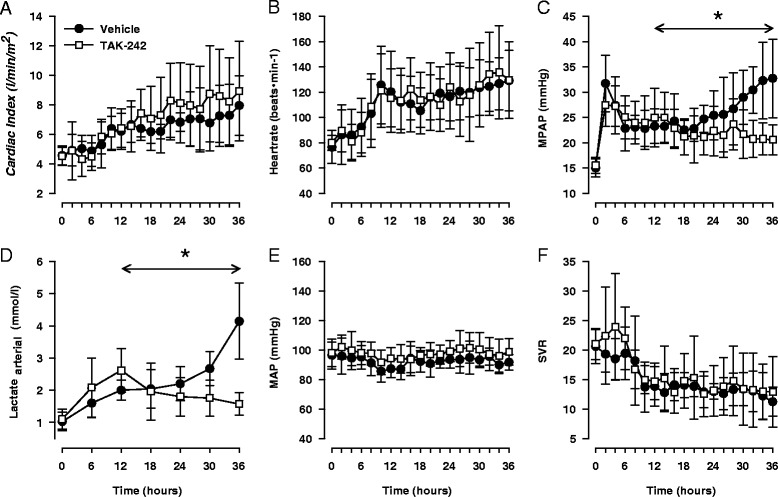
**Changes in cardiac index (A), heart rate (B), mean pulmonary arterial pressure (MPAP) (C), arterial lactate (D), mean arterial blood pressure (MAP) (E), and systemic vascular resistance (SVR) (F) in response to sepsis and treatment with either the selective TLR4 inhibitor TAK-242 (n = 7) or vehicle (n = 7).** Data are expressed as mean and 95% confidence interval. Asterisk indicates a significant difference between TAK-242 and control in response to sepsis. Analysis of variance (ANOVA) repeated measures from 12 to 36 hours.

Intravenous infusion of live *E. coli* bacteria caused classic signs of hyperdynamic sepsis; tachycardia, pulmonary hypertension, increased cardiac index, and a decrease in systemic vascular resistance (Figure 1A-C and F, *P* <0.05 for all). However, mean arterial blood pressure remained close to baseline levels, indicating that the animals were not in shock (Figure 1E). TAK-242 attenuated the increase in mean pulmonary arterial pressure (MPAP) but had no significant effect on any other of these variables (Figure 1). Arterial levels of lactate increased as a response to sepsis. However, in parallel with an improved renal function, TAK-242 reversed this trend and reduced lactate levels to near baseline values (Figure 1D). Treatment with TAK-242 attenuated the decline in arterial partial pressure of oxygen (pO_2_) caused by sepsis and it ended significantly higher compared with the control group. Partial pressure of carbon dioxide (pCO_2_) increased in both groups without intergroup difference. No significant change over time or difference between the groups was observed in arterial pH, base excess, hematocrite, plasma protein, sodium, or potassium levels (Additional file 3: Table S1). Sepsis induced nitric oxide (NO) formation as indicated by increased plasma levels of NOx (sum of nitrite and nitrate) and cyclic guanosine monophosphate (*P* <0.05 for both) with no effect of treatment with TAK-242. NOx in urine did not change significantly during the experiment (Additional file 3: Table S1).

### Sepsis-induced renal dysfunction was reversed by the TLR4 inhibitor TAK-242

**Figure 2:**
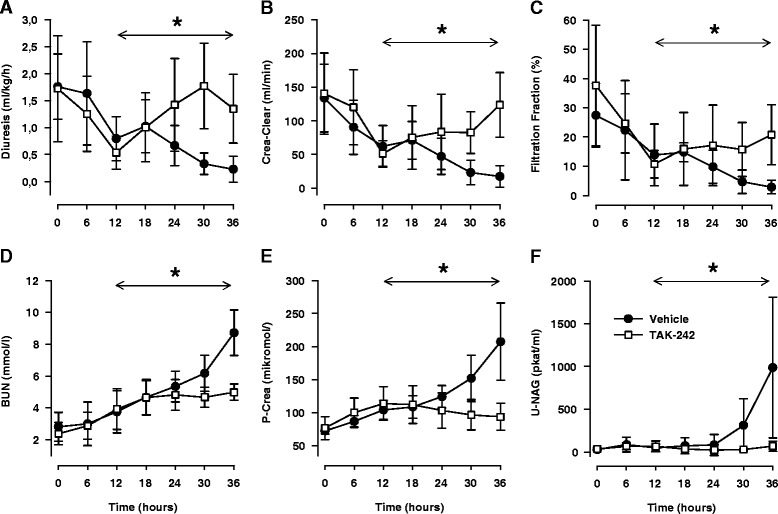
**Changes in diuresis (A), creatinine clearance (Crea Clear) (B), filtration fraction (C), blood urea nitrogen (BUN) (D) plasma creatinine (P-Crea) (E), and urinary concentrations of N-acetyl-ß-D-glucosaminidase (U-NAG) (F) in response to sepsis and treatment with either the selective TLR4 inhibitor TAK-242 (n = 7) or vehicle (n = 7).** Data are expressed as mean and 95% confidence interval. Asterisk indicates a significant difference between TAK-242 and control in response to sepsis. Analysis of variance (ANOVA) repeated measures from 12 to 36 hours.

After 12 hours of sepsis, renal function was reduced as measured by elevated plasma levels of creatinine and BUN, together with a decrease in creatinine clearance, urine production, and filtration fraction (Figure 2A-E, *P* <0.05 for all). The TLR4 inhibitor had a clear beneficial effect on renal function: Urine production decreased progressively in the control group, reaching severe oliguria in all animals, but TAK-242 not only prevented this decline but also improved diuresis significantly between 12 and 36 hours (*P* <0.01, Figure 2A). Furthermore, treatment with TAK-242 abolished the impairment in creatinine clearance (Figure 2, *P* = 0.002) and attenuated the increase in plasma levels of creatinine, filtration fraction, and BUN (*P* <0.05, for all Figure 2). U-NAG, a marker of tubular damage, was significantly elevated at the end of the experiment, but TAK-242 attenuated this increase (*P* <0.05, Figure 2F), indicating less tubular injury in sheep with TLR4 inhibition.

### Sepsis did not cause renal hypoperfusion

**Figure 3:**
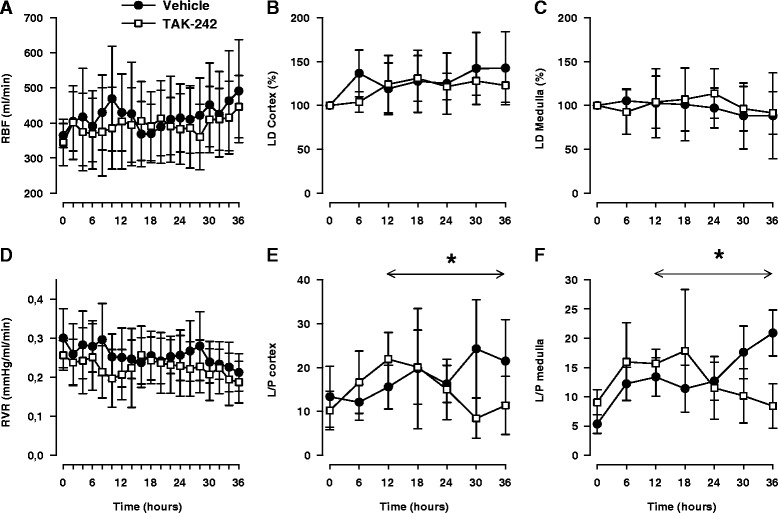
**Changes in renal artery blood flow (RBF) (A), laser Doppler changes in renal cortex (LD Cortex) (B), laser Doppler changes in renal medulla (LD Medulla) (C), renal vascular resistance (RVR) (D), lactate/pyruvate ratio in cortex (L/P Cortex) (E), and lactate/pyruvate ratio in medulla (L/P Medulla) (F) in response to sepsis and treatment with either the selective TLR4 inhibitor TAK-242 (n = 7) or vehicle (n = 7).** Data are expressed as mean and 95% confidence interval. Asterisk indicates a significant difference between TAK-242 and control in response to sepsis. Analysis of variance (ANOVA) repeated measures from 12 to 36 hours.

**Figure 4:**
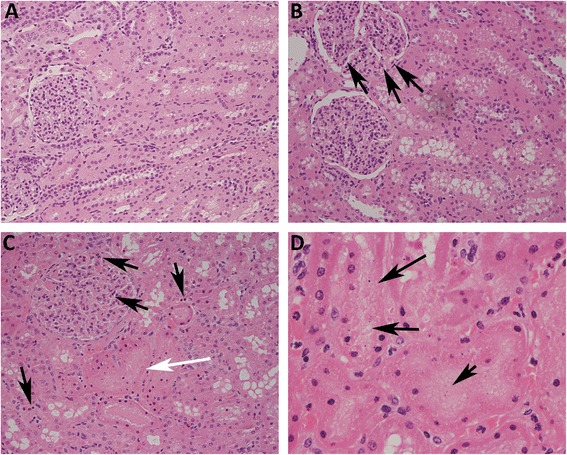
**Increased renal neutrophil infiltration is attenuated by TLR4 inhibition.** Light microscopy with hematoxylin/eosin staining shows an increased number of polymorphonuclear leukocytes (PMNs) (arrows) in the glomeruli and within the interstitium/peritubular capillaries in both vehicle-treated **(C)** and TAK-242-treated **(B)** animals in comparison with sham **(A)**. Tubular epithelial injury was seen with pyknotic and disintegrated nuclei in two of the vehicle-treated animals—**(C)** white arrow and **(D)** arrows—and one of the TAK-242-treated animals.

A possible explanation for the beneficial effects of TAK-242 was prevention of renal hypoperfusion. However, renal artery blood flow increased (*P* <0.001) during sepsis and renal vascular resistance was significantly decreased (*P* <0.001) without any intergroup differences (Figure 3). This was mirrored as an increased perfusion in the renal cortex (*P* <0.05), but medullary perfusion remained close to baseline levels in both groups (Figure 3). Light microscopy revealed no obvious renal tubular damage that explained the renal dysfunction. The tubules appeared normal except in two of the vehicle animals, where areas of proximal tubular injury were seen within the cortex (Figure 4). In such areas, the epithelial cells showed pyknotic or disintegrated nuclei (Figure 4D) or isometric vacuolization of the cytoplasm or both. In one of the TAK-242-treated animals, similar but much milder changes were seen. No changes were seen in larger vessels. In two of the vehicle animals, fibrin thrombi were found in the glomerular capillaries (Additional file 4: Figure S1).

### Treatment with a TLR4 inhibitor attenuated septic renal hyperlactemia

To measure renal metabolic changes, microdialysis probes were placed in the renal cortex and medulla. During the first 12 hours of sepsis lactate, pyruvate and the lactate/pyruvate (L/P) ratio increased in both the cortex and the medulla whereas glucose was not significantly altered (Additional file 5: Table S2). TAK-242 significantly attenuated the increase in lactate (Additional file 5: Table S2) and reduced the L/P ratio (Figure 3) in both cortex and medulla. However, glucose and pyruvate levels remained stable, and no effect of treatment was observed (Additional file 5: Table S2).

### Treatment with a TLR4 inhibitor reduced renal neutrophil infiltration

**Figure 5:**
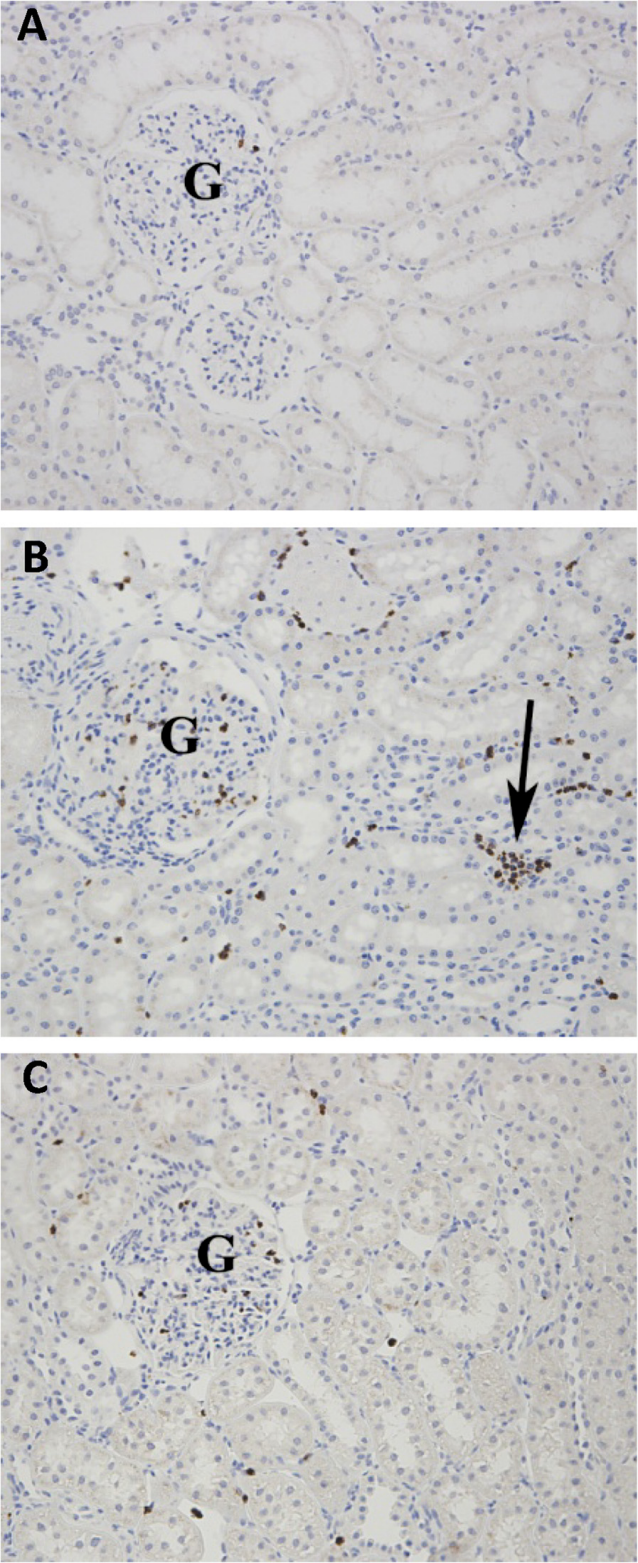
**Immunohistochemistry with anti-myeloperoxidase antibodies show increased number of polymorphonuclear leukocytes (PMNs) in glomerular capillaries (G) and interstitium/peritubular capillaries (arrow) in vehicle-treated (B) and TAK-242-treated (C) group in comparison with sham (A).**

**Figure 6:**
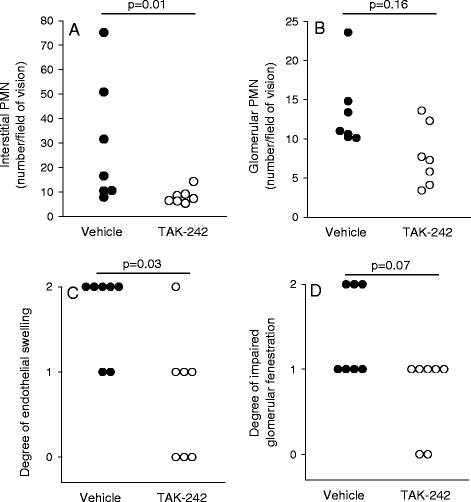
**Number of polymorphonuclear leukocytes (PMNs) in interstitium (A) and glomerulus (B) as well as histological determination of endothelial swelling (C) and degree of fenestration in vehicle-treated (D) (n = 7) and TAK-242-treated (n = 7) animals.** Zero indicates no swelling/decreased fenestration, 1 indicates some swelling/decreased fenestration, and 2 indicates severe swelling/decreased fenestration as determined by electron microscopy. Data are expressed as raw numbers. *P* <0.05 is considered significant.

In the vehicle- and TAK-242-treated groups, increased amounts of PMN cells were found in the glomeruli and the interstitium/peritubular capillaries compared with controls. This was confirmed by quantification of PMNs by using immunohistochemistry (Figure 5). Compared with vehicle, TAK-242 significantly reduced the number of PMNs in the interstitium (*P* = 0.01, Figure 6A) but not in the glomerulus (*P* = 0.16, Figure 6B).

### Sepsis-induced endothelial swelling and decreased fenestration in the glomeruli were attenuated by a TLR4 inhibitor

**Figure 7:**
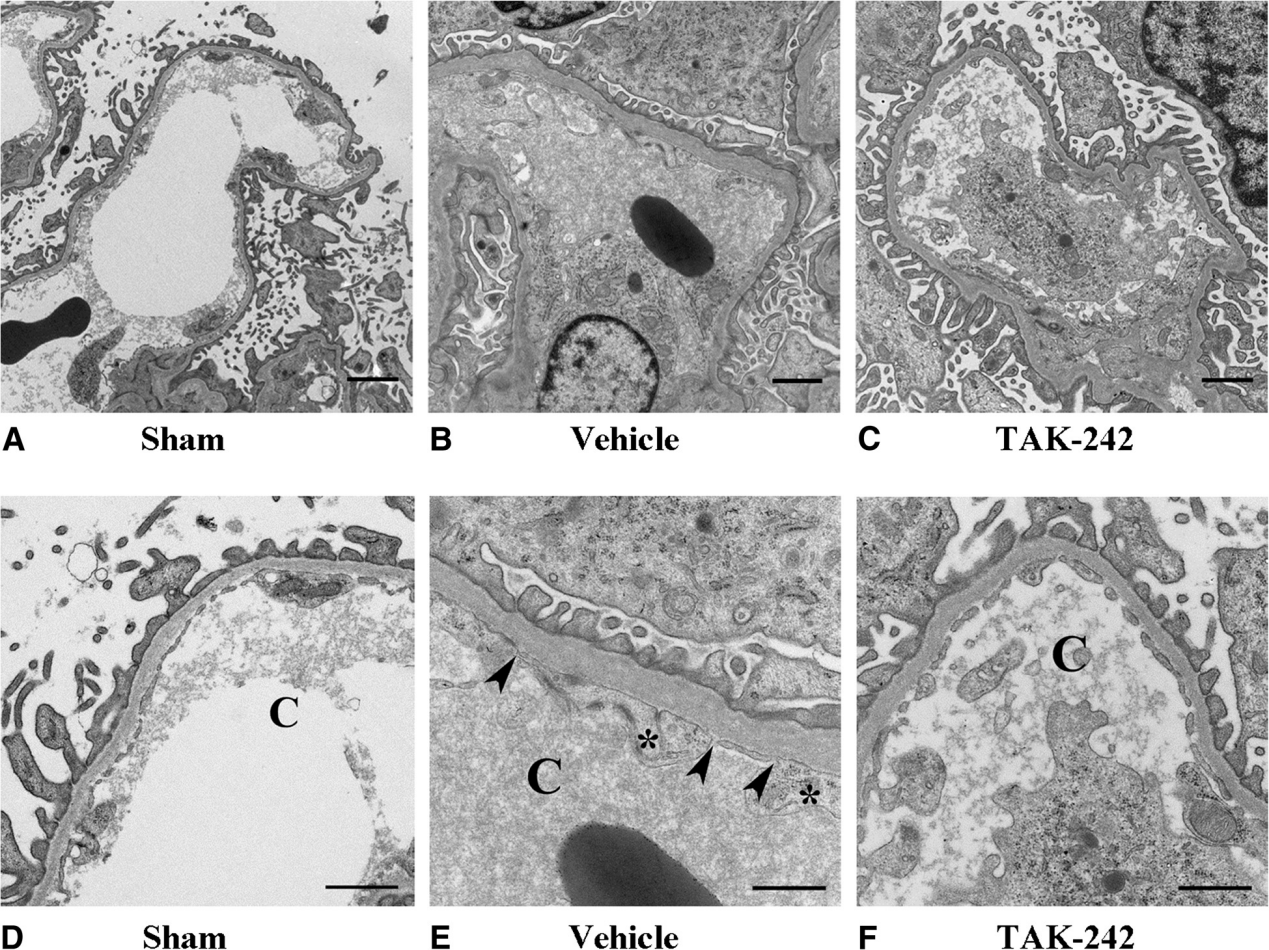
**Electron microscopic images of the glomerular capillaries in sham (A), vehicle-treated (B), and TAK-242-treated (C) animals.** The same images magnified showing glomerular capillaries in sham **(D)**, vehicle-treated **(E)**, and TAK-242-treated **(F)** animals. Asterisk indicates endothelial cell swelling, and arrowheads show decreased fenestration of the glomerular endothelium. Bars = 2 μm **(A, B, C)** and 1 μm **(D, E, F)**.

To examine the glomerular effects of sepsis and subsequent treatment with a TLR4 inhibitor, electron microscopy was used. There was no difference in the degree of foot process effacement or in the thickness of the glomerular basement membrane (GBM) between the vehicle-treated (232 ± 29 nm), TAK-242-treated (264 ± 72 nm), and sham (242 ± 9 nm) groups (Figure 7). However, there was a difference in the ultrastructure of the endothelial cells in the glomerular capillaries. The endothelial cells of the vehicle-treated animals were more swollen (*P* = 0.03, Figures 6C and 7) and showed a tendency for decreased fenestration (*P* = 0.07, Figures 6D and 7) compared with TAK-242-treated animals and sham. Furthermore, the light microscopic observation of fibrin thrombi was confirmed in the two vehicle-treated animals (Additional file 4: Figure S1).

### TAK-242 had no effect per se

There were no obvious differences in MAP, HR, CI, or MPAP between TAK-242 and vehicle during the 12-hour treatment period in healthy sheep (Additional file 6: Figure S2). Renal function, as evaluated by creatinine clearance, plasma clearance, BUN, and urine output, remained stable, and no difference between treatments was seen (Additional file 7: Figure S3). Finally, renal blood flow and blood gas parameters remained unaffected by TAK-242 per se (Additional file 7: Figure S3 and Additional file 8: Table S4).

## Discussion

In the present study, we aimed to investigate the renal effects of a TLR4-inhbitor, TAK-242, in experimental sepsis. The most striking finding was that TAK-242 reversed a progressive decline in renal function when administered therapeutically after 12 hours of hyperdynamic *E. coli* sepsis. This was associated with a prominent reduction in renal neutrophil infiltration, a decreased swelling of the endothelium in the glomerular capillaries, an attenuation of arterial and renal hyperlactemia, and less tubular damage as indicated by reduced U-NAG. Furthermore, no major effects on systemic or local renal hemodynamics by TAK-242 were seen during sepsis, and in separate experiments in animals without sepsis, TAK-242 had no obvious major effects per se.

Proper animal models of septic AKI are difficult to achieve, and too often they do not mimic the clinical scenario [24]. In this study, we used fluid-resuscitated, conscious animals developing severe hyperdynamic sepsis caused by live bacteria. Detailed renal functional data were collected repeatedly over an extended time period and related to immune function and histological pathology. In addition, treatment was started when sepsis and renal dysfunction were already present. Another strength of this investigation was that the renal histological findings in large mimicked what was recently described in human kidney after sepsis [6].

Innate immunity is the first line of defense against invading microbes and is crucial for preventing infections. TLR4 is central in this immune activation by binding LPS [9,25], which results in an inflammatory response that induces the production and release of cytokines as well as stimulation of inflammatory cells [11]. Among these cells are neutrophils, which are activated and stimulated to transmigrate from blood to tissue. This is caused by TLR4-mediated release of interleukin-1B (IL-1B), tumor necrosis factor-alpha (TNFα), and IL-6. Another important neutrophil activator released by TLR4 stimulation is serum amyloid protein 3, which mediates its effect by targeting the formyl peptide receptor [26]. Although the inflammatory response is crucial for preventing infections, a strong activation of the innate immune system, like in sepsis, may inflict damage to endogenous tissue and impair organ function. TAK-242 binds selectively to the intracellular domain of TLR4 and disrupts the interaction with several adaptor molecules. This leads to inhibition of both the MyD88-dependent and the TRIF (TIR domain-containing adapter-inducing IFN-β)-dependent pathway for TLR4 signal transduction [27].

Acute tubular necrosis (ATN) caused by ischemia has been assumed to be the underlying pathophysiology of septic AKI [2,28]. Massive release of NO is believed to cause vasodilation that result in relative hypovolemia, a reduction in cardiac output, and hypotension. As a reflex, renal sympathetic nerve activity is increased together with elevated levels of vasopressin, endothelin, angiotensin II, and aldosterone. The resulting renal vascular vasoconstriction leads to ischemia and subsequent ATN that impairs renal function. Undoubtedly, ischemia is a crucial factor for many types of AKI. However, in sepsis, the situation may sometimes be different. Most patients with sepsis present with a hyperdynamic circulation with elevated cardiac output [29]. Experimental data indicate that AKI develops although total renal blood flow is increased [4,23] and hypotension is counteracted by vasoactive drugs [3]. Furthermore, the correlation between ATN and septic renal failure is weak in both human and animal studies [30]. The current results obtained in normotensive septic sheep confirm that hypotension is not a necessary attribute for septic AKI. This is in line with studies in critically ill patients with severe sepsis, in whom blood pressure levels did not correlate with the severity of renal failure [31]. TAK-242 drastically improved renal function without major hemodynamic effects. Moreover, there was no sign of renal vasoconstriction, as total renal artery blood flow as well as cortical and medullary perfusion did not decline during the 36-hour experiment. Thus, renal hypoperfusion appears highly unlikely as a cause of septic AKI, given this experimental setting. A recently promoted hypothesis for sepsis-induced AKI is that the inflammatory response causes a preferential dilatation of renal efferent arterioles, increasing renal blood flow but reducing the hydrostatic pressure for glomerular filtration and thereby glomerular filtration rate (GFR) [32]. The most important vasodilator released in sepsis is NO, and it has been suggested that excessive intra-renal NO could be responsible for the reduction in post-glomerular resistance [33]. However, it is unlikely that the effect of TLR4 inhibition on renal function in this study is mediated by reduced NO formation as there were no differences in either MAP or renal blood flow and as TAK-242 did not affect NOx or cyclic guanosine monophosphate (cGMP) levels. This view is supported by results demonstrating no effect on renal function after intrarenal NO synthase inhibition during Gram-negative sepsis [33].

Both cortical and medullary ratios of L/P were significantly increased by sepsis but subsequently reduced by TAK-242. In view of the preserved renal circulation, the renal hyperlactemia cannot easily be explained by reduced renal oxygen supply. However, direct measurement of renal tissue oxygenation or the utilization of oxygen by renal mitochondria was not performed in this study. Elevated lactate levels in sepsis not related to hypoxia have been linked to stimulation of muscle Na/K-ATPase [34] and mitochondrial dysfunction [35]. Thus, besides hypoxia, other factors may explain the renal hyperlactemia.

Sepsis caused severe endothelial swelling and decreased glomerular fenestration, both of which were reduced by TLR4 inhibition. The renal dysfunction observed may, to a large extent, be a consequence of decreased glomerular filtration per se, as creatinine clearance and the filtration fraction decreased significantly in the vehicle-treated animals, without significant tubular damage. Endothelial dysfunction in the peritubular capillaries has been highlighted as an important injury pathway in septic AKI [36], possibly by inducing ATN [37]. Herein, U-NAG did increase in vehicle-treated animals as a possible sign of tubular injury, but this took place several hours after renal function started to decline and was not confirmed by light microscopy. Instead, it is possible that endothelial swelling and perhaps decreased fenestration in the glomerulus may play an important role in the injury pathway and propagation of septic AKI. However, an early and specific marker of glomerular membrane dysfunction is urine protein leakage, and this was not detected in this study and is not a hallmark of sepsis-induced AKI.

Mobilization and recruitment of PMNs by the innate immune defense constitute a key event in response to an infection. After extravasation, PMNs destroy invading organisms by phagocytosis, release of acid hydrolases and antimicrobial peptides, and stimulation of antibiotic actions of monocytes and macrophages [38]. However, PMN degranulation may also inflict damage to endogenous tissue. The role of PMNs in septic AKI is largely unknown, but after renal ischemia and reperfusion, PMNs have been shown to transmigrate from the circulation and contribute to AKI [39]. In sepsis, Castoldi and colleagues [40] recently showed that TLR4-deficient mice had reduced renal neutrophil activation and infiltration compared with wild-type mice and that neutrophil depletion improved renal function. This indicates that the extensive renal neutrophil accumulation caused by sepsis in this study is potentially deleterious. Activation of renal endothelial TLR4 has been suggested to play a critical role in the upregulation of adhesion molecules which can promote the recruitment of leukocytes to areas of injury and aggravate damage and inflammation in the tissue [41,42]. A TLR4-dependent pathway promoting renal injury and inflammation in antibody-mediated glomeruli-nephritis and cisplatin-induced nephrotoxicity has also been described [43,44]. Therefore, reduced TLR4-dependent infiltration of PMNs may have contributed to the improved renal function in the TAK-242 group. This is supported by recent findings of substantial renal leukocyte infiltration in human septic shock [6]. Possible mechanisms for the renal dysfunction include PMN vascular endothelial attachment that reduces blood flow and causes ischemia [45] or tubular damage by migrated PMNs [46]. However, as discussed previously, no rheological effects of sepsis or TLR4 inhibition were noted in the current study, and light microscopy revealed no convincing evidence of general or widely spread structural damage to the tubules. Taken together, these observations indicate that neutrophil activation and recruitment into the kidney are potential and perhaps crucial mediators of septic AKI, although the underlying mechanism remains unknown. As shown in a series of elegant experiments by Watts and colleagues [47-49], TLR4 activation may also affect renal function by impairing tubular transport. In particular, HCO_3_^−^ reabsorption is inhibited by the direct effect of LPS on TLR4, and this may contribute to sepsis-induced acidosis.

Besides renal effects, TLR4 inhibition significantly attenuated the increase in mean pulmonary artery pressure and prevented the decrease in partial pressure of oxygen. The mechanism for these findings is unknown. However, a TLR4-dependent pathway for recruitment of neutrophils into the lung has been highlighted in endotoxemic mice [41], and PMN degranulation may cause severe lung damage [38]. The reason why arterial pCO_2_ did not change is unknown but may be related to the fact that CO_2_, compared with oxygen, more easily diffuses between blood and alveolus.

The current results are partly in contrast to the recent clinical trial using a TLR4 antagonist in sepsis [19,20]. In that study, no effect on survival was discovered. However, data on renal function were not reported, and only a minority of the patients had verified Gram-negative sepsis. Thus, it is possible that some patients with sepsis would still benefit from TLR4 inhibition.

We would like to acknowledge some of the limitations of this study. Evaluation of the renal microcirculation was performed with the use of laser Doppler probes surgically implanted in both the cortex and medulla. This is a well-established method and is frequently used for assessment of renal tissue perfusion; however, the technique is invasive, and measurements are performed at a small proportion of the kidney, less than approximately 1 mm^3^ at each probe site. Although the technique is a good option for continuous data acquisition [50], it has problems detecting heterogeneity in microvascular flow. The use of creatinine clearance as an indicator for GFR is often used in the clinical setting, but the accuracy is limited in patients. However, in sheep, renal clearance of endogenous creatinine may be a relatively adequate measure of GFR [51].

## Conclusions

In summary, our data show that TLR4 signaling is important for the development of septic AKI and that treatment with a TLR4 inhibitor is able to reverse a manifest reduction in renal function caused by sepsis. Although further studies are necessary, this suggests that targeting TLR4 may be a potential way to treat AKI caused by Gram-negative sepsis. Furthermore, in the present model, septic AKI is not due to hypoperfusion-induced ischemia but rather an inflammatory reaction within the kidney reducing glomerular filtration. Endothelial swelling in the glomerulus may be a contributing factor for the latter.

## Key messages

- Inhibiting Toll-like receptor 4 reverses a manifest renal dysfunction caused by *E. coli* sepsis.
- In the present model, septic acute kidney injury is not due to hypoperfusion-induced ischemia but rather an inflammatory reaction within the kidney.
- Endothelial swelling in the glomerulus may be a contributing factor for the reduced glomerular filtration.
- Toll-like receptor 4 antagonism has no obvious effects on renal function in healthy individuals.

## Additional files

Additional file 1:
**Detailed Materials and Methods.** A more extensive description of materials and methods. Additional file 2: Table S3. Sham-operated animal without sepsis. Data are for sham animal without sepsis (n = 1) and expressed as raw data from baseline to 36 hours. CI, cardiac index; cort, cortex; Crea clearance, creatinine clearance; HR, heart rate; Lactate_art_, arterial levels of lactate; LD, laser Doppler; L/P, Lactate/Pyruvate ratio; MAP, mean arterial pressure; med, medulla; MD, microdialysis; MPAP, mean pulmonary arterial pressure; pCO_2_, partial pressure of carbon dioxide; pO_2_, partial pressure of oxygen; P-Protein, plasma protein; RBF, renal blood flow. Additional file 3: Table S1. Systemic variables. Data are for TAK-242 and control expressed as mean and standard deviation (SD). Asterisk indicates a significant difference between TAK-242 and control in response to sepsis. Analysis of variance (ANOVA) repeated measures, including 12, 18, 24, 30, and 36 hours. Differences were considered significant at *P* ≤0.05. cGMP, cyclic guanosine monophosphate; NOx, the sum of nitrite and nitrate concentration; pCO_2_, partial pressure of carbon dioxide; pO_2_, partial pressure of oxygen; p-protein, plasma protein. Additional file 4: Figure S1. Fibrin thrombi formation in glomerular capillaries. Fibrin thrombi formation in glomerular capillaries. In two of the vehicle-treated animals, fibrin thrombi (arrow) were seen both in light microscopy with Ladewig staining (a) and in electron microscopy (b). Additional file 5: Table S2. Microdialysis in renal cortex and medulla. Data are for TAK-242 and control expressed as mean and standard deviation (SD). Asterisk indicates a significant difference between TAK-242 and control in response to sepsis. Analysis of variance (ANOVA) repeated measures, including 12, 18, 24, 30, and 36 hours. Differences were considered significant at *P* ≤0.05. cort, cortex; MD, microdialysis; med, medulla. Additional file 6: Figure S2. Changes in heart rate (A), cardiac index (B), mean pulmonary artery pressure (MPAP) (C), and mean arterial pressure (MAP) (D) in response to treatment with either the selective TLR4 inhibitor TAK-242 (filled symbols) or vehicle (open symbols). N = 3 in a cross-over design. Data are shown for each individual animal. Additional file 7: Figure S3. Changes in creatinine clearance (A), P-creatinine (B), blood urea nitrogen (BUN) (C), renal blood flow (D), and urine output (E) in response to treatment with either the selective TLR4 inhibitor TAK-242 (filled symbols) or vehicle (open symbols). N = 3 in a cross-over design. Data are shown for each individual animal. Additional file 8: Table S4. Sheep without sepsis treated with TAK-242. Blood gas data for three healthy sheep receiving TAK-242 or vehicle for 12 hours in a cross-over design.

## Abbreviations

AKI: acute kidney injury
ANOVA: analysis of variance
ATN: acute tubular necrosis
BUN: blood urea nitrogen
CI: cardiac index
GBM: glomerular basement membrane
GFR: glomerular filtration rate
HR: heart rate
L/P: lactate/pyruvate
LPS: lipopolysaccharide
MAP: mean arterial blood pressure
MPAP: mean pulmonary arterial blood pressure
NAG: N-acetyl-beta-D-glucosaminidas
NO: nitric oxide
NOx: sum of nitrite and nitrate
pCO_2_: partial pressure of carbon dioxide
PMN: polymorphonuclear leukocyte
TLR4: Toll-like receptor 4
