# Supplementary material for: Renal effects of treatment with a TLR4 inhibitor in conscious septic sheep
Source: Crit Care. 2014 Sep 3;18(5):488. doi: 10.1186/s13054-014-0488-y (PMC4190385; doi:10.1186/s13054-014-0488-y)
Supplement: Additional file 1: — Detailed Materials and Methods. A more extensive description of materials and methods. [file 13054_2014_488_MOESM1_ESM.pdf]

Material and Methods

The experiments were conducted in accordance with "The European Convention for Protection of Vertebrate Animals used for Experimental and other Scientific Purposes" (Council of Europe No 123, Strasbourg 1985). The Regional Ethics Committee for Experiments in Animals, Stockholm, Sweden approved the study in advance (N285/08).

Animals and surgical preparation

Twenty-seven adult Texel crossbred ewes were included in the study. The animals were housed individually in pens with free access to water and fed hay and commercial pellets (75 gram) with addition of 6 gram NaCl. Twenty-four animals were subjected to a surgical preparation, performed under sterile conditions, and were then allowed to recover 12-18 hours before the experiments commenced. Surgical anaesthesia included premedication with acepromazin (0.3 mg•kg^{-1} iv), anaesthesia induction with sodium thiopental injection (10 mg•min^{-1} iv) and maintenance with isoflurane (2.1-2.3 % endtidal concentration) in an O_2/air mixture (30/70) from a ventilator as previously described in detail[24]. First a small skin incision was made and the carotid artery was visualized and cannulated. Intravascular catheters were inserted in the jugular veins. A flow-directed thermodilution catheter (Swan-Ganz, Edward labs, Santa Ana, CA, USA) was fed into the pulmonary artery via an introducer in the right jugular vein and the position was determined by pressure guidance on a monitor. All catheters were sutured to the skin and the incision above the carotid artery closed. A retention catheter (14 F) was inserted into the bladder via the urethra. After a flank incision the kidney was visualized and an ultrasonic flow probe was placed around the renal artery. Thereafter a laser Doppler probe (0.25mm fiber separation, 780nm wavelength; Perimed AB, Järfälla, Sweden) was sutured to the surface of the kidney for cortical measurements and a needle laser Doppler probe (0.15mm fiber separation, 780nm wavelength) was inserted 10-12

mm into the kidney for medullary measurements. Two microdialysis catheters (CMA 20, membrane length 10mm, shaft length 14mm, diameter 0.5mm, 20,000 Dalton membrane cut-off, CMA Microdialysis, Stockholm, Sweden) was inserted into the cortex and medulla of the kidney. After surgical preparation, the flank incision was carefully closed. Connecting tubes were tunnelled subcutaneously and their connectors placed at a paralumbar position. Postoperative fluid support was given with hydroxyethyl starch, 10 ml•kg^{-1}, iv (130/0.4, 60 mg/ml, Voluven, Fresenius Kabi, Uppsala, Sweden) and with Ringer´s Acetate solution (B. Braun Melsungen, AG, Melsungen, Germany) iv at 1 ml·kg^{-1}·h^{-1}. Peri- and postoperative analgesics were administered as buprenorphine (0.002 mg•kg^{-1} im) and fentanyl (1 µg•kg^{-1} iv).

Microdialysis

The two microdialysis probes were continuously perfused (CMA 402 syringe pump, CMA Microdialysis) with a perfusion solution (0.3µl•min^{-1}) (T1 solution, 147 mM Na^+, 4 mM K^+, 2.3 mM Ca^{2+}, and 156 mM Cl^-, CMA Microdialysis) . A recovery period of 12-18 hours was allowed before baseline measurements. Every sample period was 6 hours and adjustment for time delay of samples reaching the vials was done. All samples were analyzed immediately with regards to glucose, lactate and pyruvate on a bench top analyzer (CMA 600 microdialysis analyzer, CMA Microdialysis).

Hemodynamic measurements

Hemodynamic measurements were acquired online with a sampling rate of 50 Hz via a multichannel interface (MP150, Biopac Systems, Goleta, CA) with acquisition software (AcqKnowledge 3.8.1.; Biopac Systems). The different blood pressures were measured via pressure transducers (DPT-6003, PVB Medizin Technik, BMBH, Kirchseen, Germany). These were calibrated to atmospheric pressure at the level of the heart and to 100 mmHg or 25 mmHg using a saline column. The two Laser Doppler probes were connected to a Periflux

5001 base unit (780nm wavelength, 15 kHz band width, 0.2-s time constant; Perimed AB). Calibration was performed according to the manufacturer's instructions. The pulmonary artery catheter was connected to a Vigilance Monitor system (Edwards Lifesciences) where the cardiac output was calculated every 30-60 seconds and core temperature measured continuously. When the body temperature exceeded the upper range for automatic measurements (*i.e* 41 degrees Celsius), manual cardiac output measurements were performed by three consecutive injections (10 ml) of isotonic saline. Mean arterial- (MAP) and mean pulmonary arterial pressures (MPAP) were calculated and displayed on-line.

Blood, plasma and urine analyses

The blood samples were immediately centrifuged at + 4°C (3000 rpm) for ten minutes in pre chilled tubes with heparin or EDTA as anticoagulants. Plasma aliquots were stored at –20°C until assayed for blood urea nitrogen (Synchron LX, Beckman Instruments, Richmond, CA) and creatinine (Jaffe method). Plasma was also used for determination of hematocrite and protein concentration by refractometry (Atago Co, Tokyo, Japan). Protein levels in the urine were measured using Bradford analysis. The carotid blood samples were used for immediate arterial blood gas analyses (ABL 77, Radiometer, Copenhagen, Denmark). In addition, lactate (Accu-trend Lactate, Roche Diagnostics, Basel, Switzerland) were analysed using arterial blood. Urinary concentrations of N-acetyl-ß-D-glucosaminidase (U-NAG) were measured by a colorimetric assay (Cobas Mira, Hoffmann–La Roche AG, Basel, Switzerland).

For NOx-analyses (sum of nitrite and nitrate)urine samples were diluted with carrier solution (10% methanol) and plasma samples were extracted using methanol. Nitrite and nitrate levels were measured by a sensitive and selective measurement, high performance liquid chromatography system (ENO-20 Eicom Japan), which uses reverse phase chromatography to separate nitrite from nitrate and then nitrate is reduced to nitrite through a reaction with

cadmium and reduced copper inside a reduction column. Reduced nitrite is then derivatized with Griess reagent and the level of diazo compounds is measured by a detector at 540 nm. Plasma concentrations of cGMP were analyzed with a commercial available ELISA-kit (Bio-track EIA-system, Amersham). Samples were prepared according to the manufacturer's instructions.

Bacterial preparation

The bacterial strain used in the study was isolated from a positive human blood culture and identified as Escherichia coli by Vitek2. The isolate was sub-cultured in Luria-Bertani broth in conical flasks and incubated at 30 °C over night at 200 rpm on rotary shaker. Then the broth was centrifuged and the pellet was re-suspended in PBS. The bacteria were counted, batched in 2 x 10^{11} bacteria/tube and frozen at −70 °C for further use.

Experimental protocol

The experiments were performed with conscious animals placed in individual pens with access to water and hay ad libitum. Sepsis was induced by an intravenous infusion of live E.coli bacteria (bolus of $3.9x10^9$ colony forming units followed by an infusion of $6.0x10^9 \cdot ml^{-1}$ colony forming units, starting at a rate of $0.2 ml \cdot h^{-1}$). The infusion rate was increased stepwise every 6 hours until reaching 4 $ml \cdot h^{-1}$ after 30 hours. After twelve hours of sepsis sheep were randomized to receive a bolus dose (2 $mg \cdot kg^{-1}$) followed by a continuous infusion (4 $mg \cdot kg^{-1} \cdot 24hours^{-1}$) of either the selective TLR4 inhibitor, TAK-242 (10 $mg \cdot ml^{-1}$) (n=7) or vehicle (n=7). The total volume of vehicle or TAK-242 were 0.6 $ml \cdot kg^{-1} \cdot 24hours^{-1}$. The treatment was blinded to the investigators and the content of the infusions revealed only after the experiments were performed. To exclude that surgery *per se* had a major impact on the results obtained in the TAK-242 and vehicle groups, an additional sheep served as time-control. This included surgical preparation, recovery and monitoring for 36 hours, but no E.coli infusion or treatment. An additional three sheep were used to evaluate the effect of TAK-242 on

cardiovascular and renal function *per se*. The sheep were surgically prepared with an ultra-sonic flow-probe around the renal artery as described above. At the day of the experiment intravascular catheters were inserted in the jugular veins. A flow-directed thermodilution catheter (Swan-Ganz, Edward labs, Santa Ana, CA, USA) was fed into the pulmonary artery via an introducer in the right jugular vein and the position was determined by pressure guidance on a monitor. A retention catheter (14 F) was inserted into the bladder via the urethra. In a cross-over design each sheep were randomized to receive either TAK-242 or vehicle for 12h. The treatment was initiated by a bolus dose (2 mg·kg-1) followed by a continuous infusion (2 mg·kg-1·12hours-1). After at least one day of recovery the experiment was repeated with the remaining treatment. Thus, the sheep served as their own control. Fluid volume support was administered as Ringer´s Acetate solution (B. Braun Melsungen, AG, Melsungen, Germany) iv at 1 $ml \cdot kg^{-1} \cdot h^{-1}$ and started six hours before the infusion of live E.coli bacteria. Blood samples (approximately 20 ml of venous blood and 1 ml of arterial blood) were drawn at baseline and every six hour after commencement of sepsis. Urinary output was measured and urine samples collected every second hour. The occurrence of AKI was determined by reduced renal function measured as decreased urine output and creatinine clearance[25]. After thirty-six hours of sepsis animals were deeply anaesthetized with sodium thiopental and terminated by an overdose of potassium chloride. The kidney was rapidly harvested and prepared for histological evaluation. The position of the Laser Doppler probes and microdialysis catheters was confirmed visually by opening the kidney post mortem. Renal biopsies were immediately frozen and stored at -70°C. If the animal was judged to be severely ill and in distress it was euthanized prior to the end of the protocol.

Histological evaluation of kidney biopsies

Small pieces of renal tissue were fixed in 4% paraformaldehyde, dehydrated and embedded in paraffin according to standard procedures. Two to three µm thick sections were cut on a

microtome and stained with Hematoxylin/eosin (HE), Periodic Acid Schiff (PAS) and Ladewig staining, respectively. All sections were evaluated blindly by the same investigator.

Immunohistochemistry (IHC)

Immunohistochemistry was performed on paraffin sections in a Bondmax system (Leica Biosystems, Melbourne, Australia). The sections were pretreated with H2 (40) EDTA buffer, pH 9 (S 3308, Dako A/S, Denmark) followed by incubation with Anti-Myeloperoxidase Antibody (MPO) (A 0398, Dako A/S, Denmark) diluted 1:3000 in H2 (40) + enhancer. Bound antibodies were detected by peroxidase /DAB.

Quantification of polymorphonuclear (PMN) cells in the glomeruli. The glomerulus closest to the cortex surface was chosen as a starting point. This glomerulus and another nine glomeruli were then chosen randomly towards the medulla. In each glomerulus, all positively stained cells were counted, using a 40 x objective.

Quantification of polymorphoneclear cells the interstitium and in peritubular capillaries. The area closest to the first glomerulus was chosen as a starting point. All positively stained cells within that field of vision were counted, using a 40 x objective. The next adjacent field of vision towards the medulla, not containing glomeruli, was then chosen. In total, 10 fields of vision were evaluated in the cortical area of each animal. All sections were evaluated blindly by the same investigator.

Electron microscopy (TEM)

The kidney was cut in thin slices and placed directly in 2% glutaraldehyde + 0.5% paraformaldehyde in 0.1M phosphate buffer, pH 7.4 and stored at 4°C. Specimens were rinsed in 0.1 M phosphate buffer, pH 7.4 and post fixed in 2% osmium tetroxide in 0.1 M phosphate buffer, pH 7.4 at 4°C for 2 hours, dehydrated in ethanol followed by acetone and embedded in LX-112 (Ladd, Burlington, Vermont, USA). Semithin sections were cut and

stained with toluidin blue O and used for light microscopic analysis. Ultrathin sections (approximately 40-50 nm) were then cut and contrasted with uranyl acetate followed by lead citrate and examined in a Tecnai 12 Spirit Bio TWIN transmission electron microscope (Fei company, The Netherlands) at 100 kV. Digital images were taken using a Veleta camera (Olympus Soft Imaging Solutions, GmbH, Münster, Germany)[26]. Specimens were evaluated blindly and images were taken in three randomly selected glomeruli. In each glomerulus, three capillaries and three mesangial matrix were randomly selected in low magnification. The thickness of the glomerular basement membrane (GBM) was measured, the degree of podocytic foot process effacement and the fenestration and thickness of the endothelial cells were evaluated with the treatments blinded to the investigator. The endothelial swelling and fenestration was classified according to scale with three grades respectively. 0 indicates no swelling/decreased fenestration, 1 indicates some swelling/decreased fenestration and 2 indicates severe swelling/decreased fenestration.

Statistical analysis

Cardiovascular parameters were averaged off-line. Creatinine clearance was calculated as [(Urine flow × Urine creatinine concentration)/plasma creatinine concentration]. Cardiac output was indexed to body surface area [$0.09 \times \text{body weight}^{0.67}$].

All statistical calculations were performed using Statistica 8.0 (Statsoft Inc., Tulsa, OK) and the graphs were created with Sigma Plot 11.0 (SPSS Inc., Chicago, IL). Data are expressed as means ± standard deviation of the mean (SD) or as mean and 95% confidence interval. Urine production, FENa and U-NAG values were transformed to follow a normal distribution by taking the logarithm of the raw data. Changes in parameters over time were analysed according to a two-way repeated measures ANOVA, with time as within effects and treatment (control/TAK-242) as between effects. If there was a significant interaction between time x treatment an additional one-way repeated measures ANOVA was performed for each

treatment to investigate if that group changed significantly over time. The result of this analysis is not displayed in the figures but only referred to in the Results section. The significance level was set at $p \leq 0.05$. Mann-Whitney U-test was used to evaluate difference in PMN-count and histological scores between treatments.
