# Supplementary material for: Renal effects of treatment with a TLR4 inhibitor in conscious septic sheep
Source: Crit Care. 2014 Sep 3;18(5):488. doi: 10.1186/s13054-014-0488-y (PMC4190385; doi:10.1186/s13054-014-0488-y)
Supplement: Additional file 2: Table S3. — Sham-operated animal without sepsis. Data are for sham animal without sepsis (n = 1) and expressed as raw data from baseline to 36 hours. CI, cardiac index; cort, cortex; Crea clearance, creatinine clearance; HR, heart rate; Lactateart, arterial levels of lactate; LD, laser Doppler; L/P, Lactate/Pyruvate ratio; MAP, mean arterial pressure; med, medulla; MD, microdialysis; MPAP, mean pulmonary arterial pressure; pCO2, partial pressure of carbon dioxide; pO2, partial pressure of oxygen; P-Protein, plasma protein; RBF, renal blood flow. [file 13054_2014_488_MOESM2_ESM.pdf]

Sham (n=1)		Baseline	6	12	18	24	30	36
MAP	(mmHg)	97	91	93	86	85	87	90
HR	(beats/min)	74	74	62	60	55	55	56
CI	($l/min/m^2$)	3,8	4	4,8	4,1	3,9	4	4,5
RBF	(ml/min)	298	280	271	242	262	225	220
MPAP	(mmHg)	15	18	14	15	15	15	15
pCO2	(kPa)	4,7	4,3	4,5	4,2	4,3	4,4	4,4
pO2	(kPa)	11,8	12	12,1	13,5	14,2	13,8	13,8
pH		7,48	7,49	7,49	7,51	7,55	7,54	7,51
Base Excess		2,9	2,3	2,7	2,1	5,8	5,5	3,8
LD Cortex	(%)	100	121	131	97	85	77	83
LD Medulla	(%)	100	87	95	87	87	98	98
$Lactate_{art}$	(mmol/l)	0,4	0,4	0,4	0,3	0,3	0,3	0,4
Temperature	(oC)	39,2	39,4	39,2	39,1	39,1	38,9	39
P-Protein	(g/l)	62	63	62	62	61	61	63
Diuresis	(ml/kg/h)	0,97	0,88	0,99	0,88	1,1	1,38	1,02
Crea clearance	(ml/min)	134	143	122	114	115	144	122
MD-$Glucose_{cort}$	(mmol/l)	1,8	3,5	3,5	3,1	3,1	3,3	3,5
MD-$Lactate_{cort}$	(mmol/l)	0,3	0,7	0,3	0,3	0,3	0,2	0,2
MD-$Pyruvate_{cort}$	(mmol/l)	25,5	43,5	31,4	30,5	24,7	17,4	16,6
L/P_{cort}		11,7	15,6	10,7	11	13,5	11,6	14,4
MD-$Glucose_{med}$	(mmol/l)	2,7	3,7	3,8	3,6	3,3	3,1	2,6
MD-$Lactate_{med}$	(mmol/l)	0,4	0,8	0,7	0,4	0,2	0,2	0,2
MD-$Pyruvate_{med}$	(mmol/l)	36,9	53,3	45,6	19,6	22	33,4	18,4
L/P_{med}		12	14,5	14,5	19	9,8	5,1	12,2
