# Supplementary material for: Renal effects of treatment with a TLR4 inhibitor in conscious septic sheep
Source: Crit Care. 2014 Sep 3;18(5):488. doi: 10.1186/s13054-014-0488-y (PMC4190385; doi:10.1186/s13054-014-0488-y)
Supplement: Additional file 3: Table S1. — Systemic variables. Data are for TAK-242 and control expressed as mean and standard deviation (SD). Asterisk indicates a significant difference between TAK-242 and control in response to sepsis. Analysis of variance (ANOVA) repeated measures, including 12, 18, 24, 30, and 36 hours. Differences were considered significant at P ≤0.05. cGMP, cyclic guanosine monophosphate; NOx, the sum of nitrite and nitrate concentration; pCO2, partial pressure of carbon dioxide; pO2, partial pressure of oxygen; p-protein, plasma protein. [file 13054_2014_488_MOESM3_ESM.pdf]

	Group	Baseline		6		12		18		24		30		36		
		Mean	SD	Mean	SD	Mean	SD	Mean	SD	Mean	SD	Mean	SD	Mean	SD	
Temperature	Control	39,0	0,3	40,5	0,6	40,6	0,4	41,2	0,4	40,9	0,3	40,8	0,5	40,7	0,5	
(°C)	TAK-242	39,0	0,2	40,0	0,4	40,2	0,4	40,1	0,5	40,2	0,6	40,2	0,7	40,3	0,7	
pO_2	Control	14,0	1,0	12,8	1,1	12,3	0,6	11,7	1,0	11,5	1,0	10,5	0,6	9,3	1,8	
(kPa)	TAK-242	13,5	1,3	12,3	1,6	11,4	1,4	11,0	1,8	11,2	1,1	11,6	1,5	11,7	1,2	*
pCO_2	Control	4,5	0,6	4,0	0,5	4,0	0,6	4,0	0,8	4,1	0,8	4,5	0,8	4,9	0,6	
(kPa)	TAK-242	4,2	1,0	3,7	0,5	4,1	0,4	4,1	0,5	4,4	0,6	4,6	0,7	4,8	0,6	
pH	Control	7,44	0,07	7,47	0,03	7,51	0,05	7,52	0,05	7,55	0,04	7,54	0,03	7,52	0,04	
	TAK-242	7,48	0,04	7,48	0,08	7,51	0,04	7,54	0,03	7,56	0,04	7,56	0,04	7,56	0,05	
Base Excess	Control	-1,1	3,5	-1,9	1,7	0,5	2,8	1,0	2,9	4,5	4,5	5,7	3,4	7,5	3,5	
(mmol/l)	TAK-242	0,7	3,4	-2,5	4,9	1,2	3,4	3,1	1,8	7,0	2,3	7,9	1,5	9,5	3,2	
Hematocrite	Control	33	5	40	6	36	6	36	5	37	9	37	9	36	8	
(%)	TAK-242	33	5	39	3	37	5	36	4	36	3	39	9	38	5	
P-Protein	Control	60	5	56	6	57	4	56	3	55	3	54	2	53	3	
(g/l)	TAK-242	59	6	58	6	53	8	52	7	51	5	52	6	50	6	
Na	Control	146	2	146	3	145	3	144	3	144	3	143	3	144	3	
(mmol/l)	TAK-242	145	1	146	3	146	2	146	2	144	2	144	2	144	2	
K	Control	3,9	0,3	3,6	0,5	4,4	0,5	4,4	1,3	4,2	1,2	3,9	0,8	3,8	0,8	
(mmol/l)	TAK-242	3,8	0,5	3,9	0,4	4,6	0,7	4,4	0,8	4,0	0,7	3,6	0,4	3,7	0,5	
U-Prot/U-Crea	Control	43,4	4,2			40,3	1,7							42,2	3,1	
(mg/mmol)	TAK-242	41,5	3,3			41,7	2,4							41,7	2,7	
P-cGMP	Control	33,3	19,7			55,5	18,6							90,1	59,0	
(pmol/ml)	TAK-242	25,7	9,3			53,0	33,5							56,1	34,4	
U-NOx	Control	24,5	15,7			33,5	16,8							24,5	24,9	
umol/L	TAK-242	27,8	23,0			34,0	15,0							24,1	14,9	
P-NOx	Control	4,9	1,2			7,2	2,0							7,5	2,0	
umol/L	TAK-242	5,7	1,2			6,9	1,7							6,5	2,0	
