# Supplementary figures and images for: Renal effects of treatment with a TLR4 inhibitor in conscious septic sheep
Source: Crit Care. 2014 Sep 3;18(5):488. doi: 10.1186/s13054-014-0488-y (PMC4190385; doi:10.1186/s13054-014-0488-y)

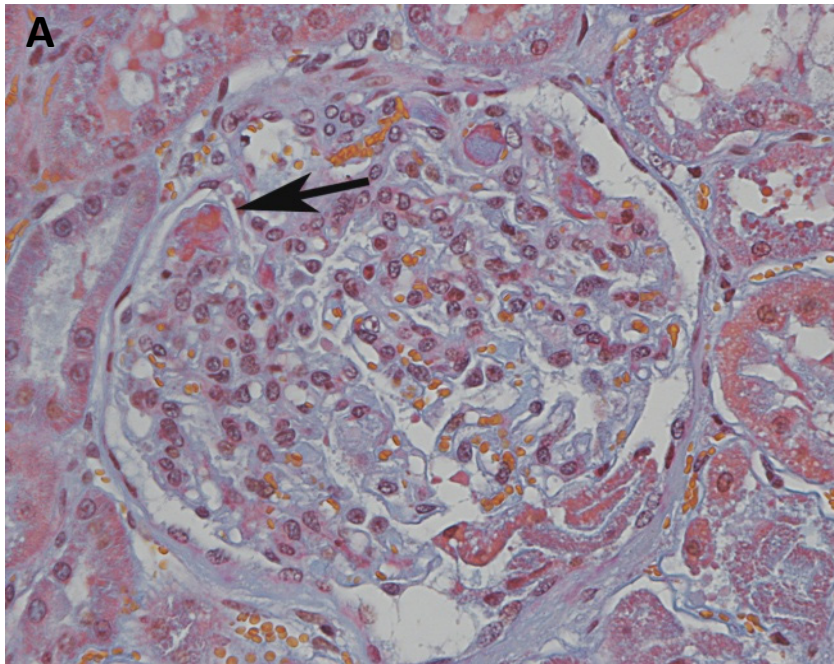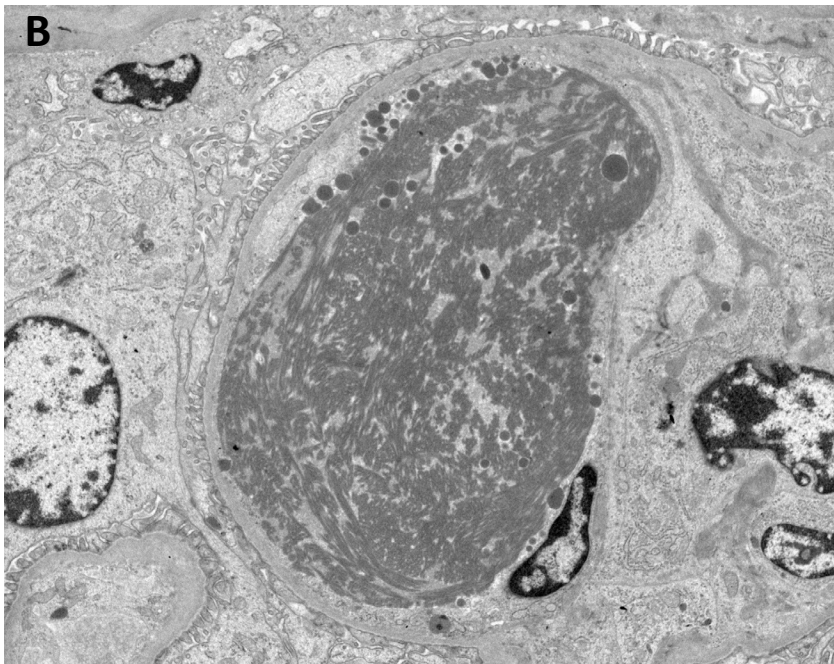

Supplement: Additional file 4: Figure S1. — Fibrin thrombi formation in glomerular capillaries. Fibrin thrombi formation in glomerular capillaries. In two of the vehicle-treated animals, fibrin thrombi (arrow) were seen both in light microscopy with Ladewig staining (a) and in electron microscopy (b). [file 13054_2014_488_MOESM4_ESM.pdf]

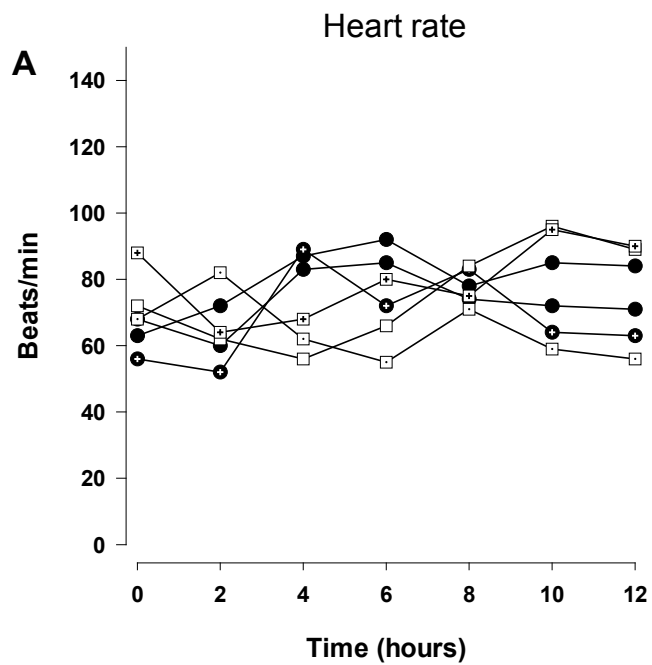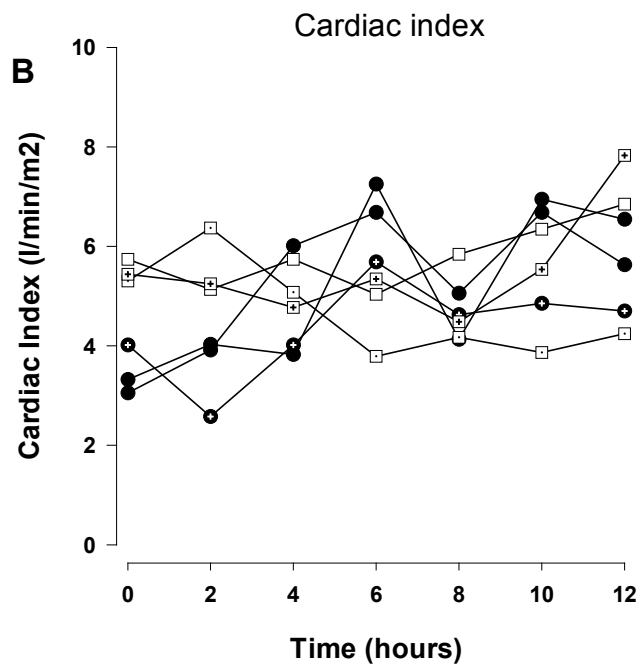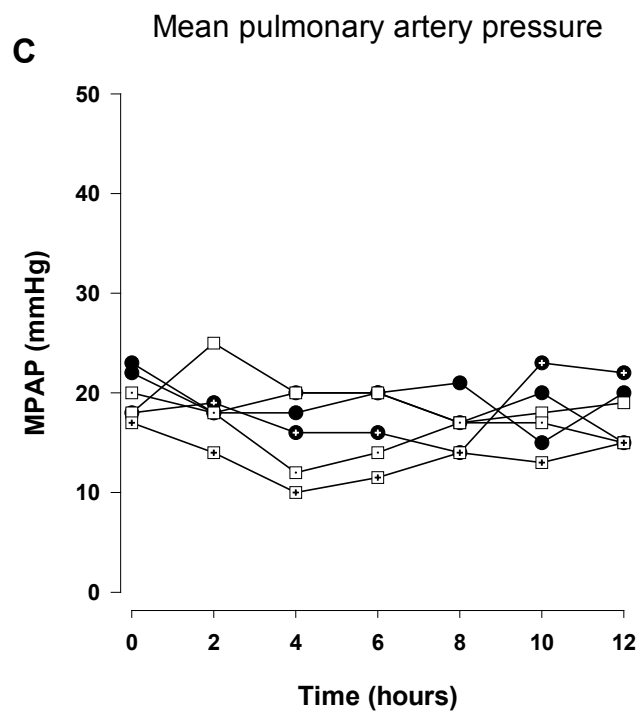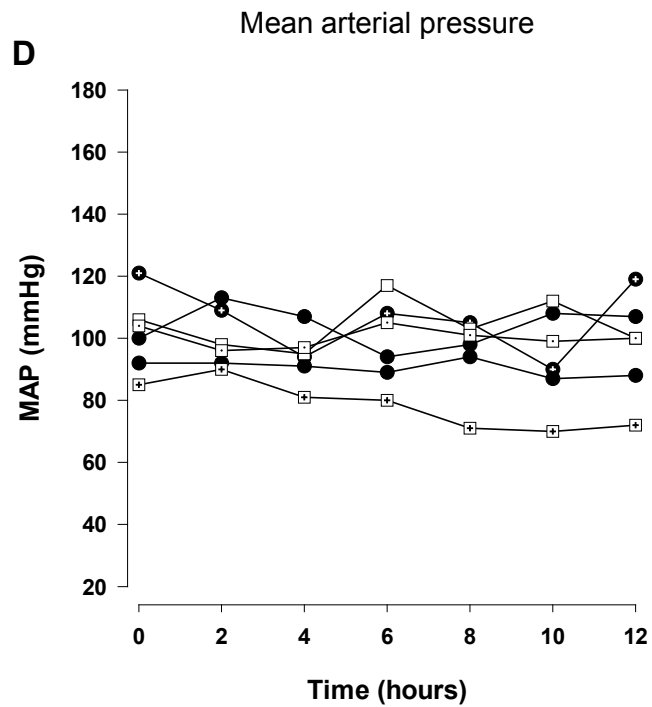

Supplement: Additional file 6: Figure S2. — Changes in heart rate (A), cardiac index (B), mean pulmonary artery pressure (MPAP) (C), and mean arterial pressure (MAP) (D) in response to treatment with either the selective TLR4 inhibitor TAK-242 (filled symbols) or vehicle (open symbols). N = 3 in a cross-over design. Data are shown for each individual animal. [file 13054_2014_488_MOESM6_ESM.pdf]

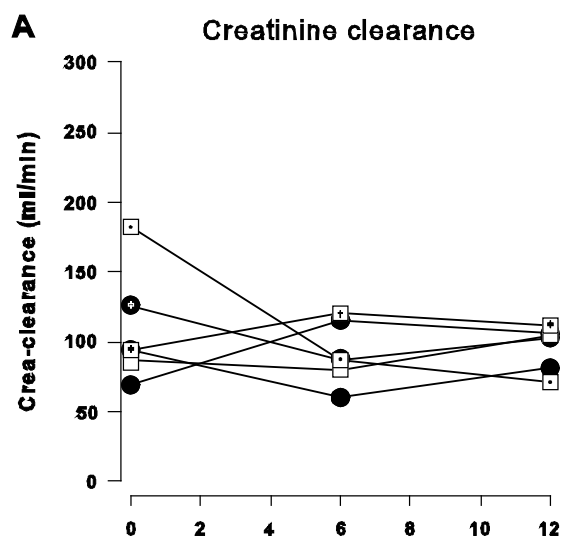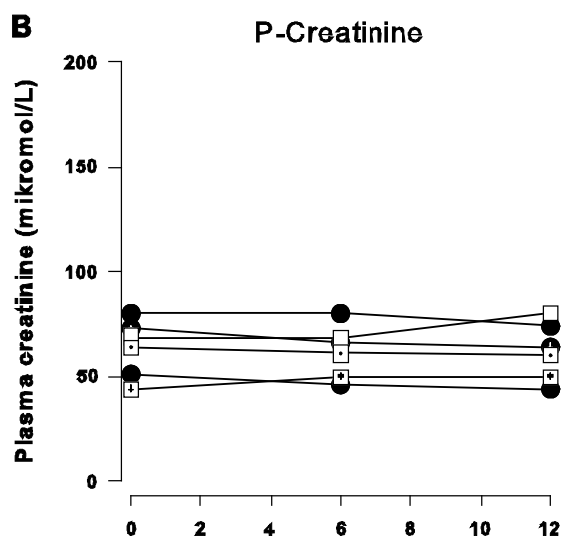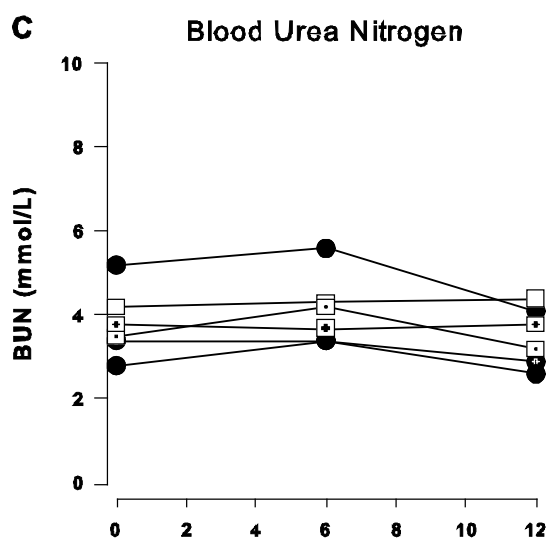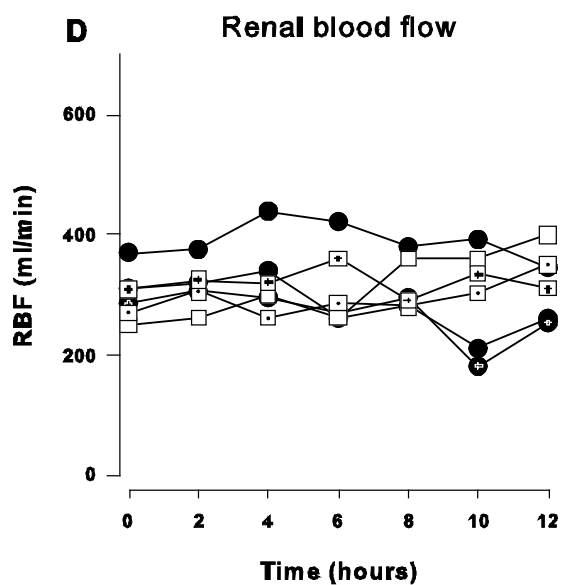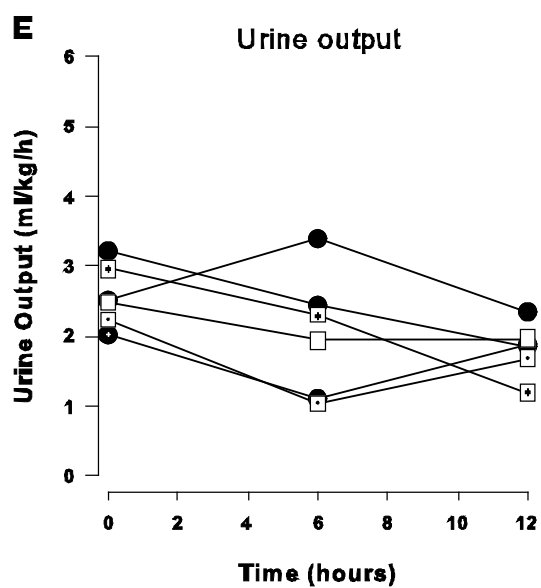

Supplement: Additional file 7: Figure S3. — Changes in creatinine clearance (A), P-creatinine (B), blood urea nitrogen (BUN) (C), renal blood flow (D), and urine output (E) in response to treatment with either the selective TLR4 inhibitor TAK-242 (filled symbols) or vehicle (open symbols). N = 3 in a cross-over design. Data are shown for each individual animal. [file 13054_2014_488_MOESM7_ESM.pdf]
