# Supplementary material for: Renal effects of treatment with a TLR4 inhibitor in conscious septic sheep
Source: Crit Care. 2014 Sep 3;18(5):488. doi: 10.1186/s13054-014-0488-y (PMC4190385; doi:10.1186/s13054-014-0488-y)
Supplement: Additional file 5: Table S2. — Microdialysis in renal cortex and medulla. Data are for TAK-242 and control expressed as mean and standard deviation (SD). Asterisk indicates a significant difference between TAK-242 and control in response to sepsis. Analysis of variance (ANOVA) repeated measures, including 12, 18, 24, 30, and 36 hours. Differences were considered significant at P ≤0.05. cort, cortex; MD, microdialysis; med, medulla. [file 13054_2014_488_MOESM5_ESM.pdf]

	Group	Baseline		6		12		18		24		30		36		
		Mean	SD	Mean	SD	Mean	SD	Mean	SD	Mean	SD	Mean	SD	Mean	SD	
MD-Glucose$_{cort}$	Control	1,4	0,3	1,5	0,4	1,4	0,1	1,5	0,2	1,4	0,3	1,4	0,3	1,6	0,3	
(mmol/l)	TAK-242	1,4	0,2	1,3	0,3	1,3	0,2	1,4	0,2	1,4	0,2	1,5	0,2	1,7	0,2	
MD-Lactate$_{cort}$	Control	0,3	0,2	0,4	0,2	0,7	0,4	1,0	0,6	1,0	0,3	1,2	0,2	1,4	0,6	
(mmol/1)	TAK-242	0,3	0,1	0,6	0,3	0,9	0,4	1,1	0,7	1,1	0,3	0,7	0,5	0,9	0,5	*
MD-Pyruvate$_{cort}$	Control	23,8	11,3	35,9	9,5	43,0	15,6	52,2	12,7	61,0	20,2	60,9	27,1	70,0	25,5	
(uM)	TAK-242	25,8	5,9	32,3	10,9	43,7	21,3	59,7	42,5	85,9	36,8	75,9	36,6	83,7	18,4	
MD-Glucose$_{med}$	Control	2,2	1,0	2,3	0,4	1,2	0,6	1,3	0,6	1,5	0,5	1,3	0,4	1,1	0,4	
(mmol/l)	TAK-242	2,1	0,6	1,9	0,5	1,7	0,6	1,7	0,5	1,5	0,4	1,6	0,3	1,4	0,6	
MD-Lactate$_{med}$	Control	0,4	0,2	1,2	0,4	1,5	0,6	1,3	0,4	1,6	0,5	1,6	0,5	1,7	0,7	
(mmol/1)	TAK-242	0,5	0,2	1,1	0,5	1,9	0,7	2,1	0,7	1,5	0,5	1,5	0,3	1,3	0,5	*
MD-Pyruvate$_{med}$	Control	82,9	43,9	100,7	38,0	105,9	21,8	124,0	45,7	132,9	44,5	88,3	12,3	83,9	40,4	
(umol/l)	TAK-242	57,1	24,6	75,8	33,9	125,0	57,6	149,2	80,9	146,8	69,6	163,9	71,1	176,8	96,9	
