# Supplementary material for: Renal effects of treatment with a TLR4 inhibitor in conscious septic sheep
Source: Crit Care. 2014 Sep 3;18(5):488. doi: 10.1186/s13054-014-0488-y (PMC4190385; doi:10.1186/s13054-014-0488-y)
Supplement: Additional file 8: Table S4. — Sheep without sepsis treated with TAK-242. Blood gas data for three healthy sheep receiving TAK-242 or vehicle for 12 hours in a cross-over design. [file 13054_2014_488_MOESM8_ESM.pdf]

Group	Variabel	Time (hours) 0	6	12
Vehicle animal 1	pH	7,50	7,43	7,45
Vehicle animal 2	pH	7,46	7,42	7,44
Vehicle animal 3	pH	7,48	7,46	7,47
TAK-242 animal 1	pH	7,47	7,53	7,51
TAK-242 animal 2	pH	7,47	7,46	7,43
TAK-242 animal 3	pH	7,54	7,49	7,47
**Average Vehicle**	pH	**7,48**	**7,44**	**7,45**
**Average TAK-242**	pH	**7,49**	**7,49**	**7,47**
Vehicle animal 1	P-Lactate (mmol/L)	0,4	0,5	0,5
Vehicle animal 2	P-Lactate (mmol/L)	0,5	0,4	0,3
Vehicle animal 3	P-Lactate (mmol/L)	1	0,8	0,7
TAK-242 animal 1	P-Lactate (mmol/L)	0,4	0,5	0,5
TAK-242 animal 2	P-Lactate (mmol/L)	0,4	0,4	0,4
TAK-242 animal 3	P-Lactate (mmol/L)	0,6	0,6	0,4
**Average Vehicle**	P-Lactate (mmol/L)	**0,6**	**0,6**	**0,5**
**Average TAK-242**	P-Lactate (mmol/L)	**0,5**	**0,5**	**0,4**
Vehicle animal 1	P-K+ (mmol/L)	3,7	4,4	4,1
Vehicle animal 2	P-K+ (mmol/L)	3,3	4,3	4,4
Vehicle animal 3	P-K+ (mmol/L)	3,6	3,9	4,3
TAK-242 animal 1	P-K+ (mmol/L)	3,4	4,5	4,4
TAK-242 animal 2	P-K+ (mmol/L)	3,4	4,1	4,3
TAK-242 animal 3	P-K+ (mmol/L)	4	4,4	4,4
**Average Vehicle**	P-K+ (mmol/L)	**3,5**	**4,2**	**4,3**
**Average TAK-242**	P-K+ (mmol/L)	**3,6**	**4,3**	**4,4**
Vehicle animal 1	P-Na+ (mmol/L)	145	146	148
Vehicle animal 2	P-Na+ (mmol/L)	147	146	147
Vehicle animal 3	P-Na+ (mmol/L)	147	147	147
TAK-242 animal 1	P-Na+ (mmol/L)	147	144	144
TAK-242 animal 2	P-Na+ (mmol/L)	147	146	147
TAK-242 animal 3	P-Na+ (mmol/L)	146	145	146
**Average Vehicle**	P-Na+ (mmol/L)	**146**	**146**	**147**
**Average TAK-242**	P-Na+ (mmol/L)	**147**	**145**	**146**
